
JOURNAL INFORMATION
==============================
NLM Title Abbreviation: Hemasphere
Journal ID: Hemasphere
NLM Journal ID: 101740619
Journal ID: HEM3

HemaSphere

EISSN: 2572-9241
Publisher: Wiley

ARTICLE INFORMATION
==============================
PMCID: PMC11663230
DOI: 10.1002/hem3.70044
Article ID: HEM370044
Article version: 1
Subjects: Issue Information, Issue Information

Issue Information

Electronic publication date: 2024 Dec 21
Publication date: 2024 Dec
Volume: 8
Issue: Suppl 3
Electronic Location ID: e70044
Copyright: © 2024 The Authors. HemaSphere published by John Wiley & Sons Ltd. on behalf of European Hematology Association.
License: This is an open access article under the terms of the http://creativecommons.org/licenses/by/4.0/ License, which permits use, distribution and reproduction in any medium, provided the original work is properly cited.
License URL: https://creativecommons.org/licenses/by/4.0/

Figure count: 0
Table count: 0
Page count: 4

source-schema-version-number: 2.0
cover-date: December 2024
details-of-publishers-convertor: Converter:WILEY_ML3GV2_TO_JATSPMC version:6.5.1 mode:remove_FC converted:22.12.2024

==============================

### HemaSphere

Vol. 8 - Issue S3

December 2024

The 18th Annual

### Sickle Cell & Thalassaemia Conference

### ABSTRACT BOOK

October 2023

ASCAT
Academy For Sickle Cell & Thalassaemia

British Society for Haematology
Listening • Learning • Leading eha

WILEY

hemaspherejournal.com eha

HemaSphere

### Aims and Scope

*HemaSphere* publishes results of highly relevant basic, translational, and clinical research in hematology. We are especially looking for strong studies reporting novel findings that are of high impact to the field of hematology.

Review articles and guideline articles provide clear overviews and discussions of new developments as well as recommendations for patient care.

*HemaSphere* is the premier hematology information resource, which is enhanced by the HemaTopics and HemaPolicy sections of the journal. These sections provide insightful discussions on all aspects related to hematology, including summaries of important articles, reports of new therapies, discussions on European policy, and other hematology news items. *HemaSphere* is guided by Editor in Chief Jan Cools and Deputy Editor in Chief Claire Harrison. The Editors and Editorial Board of *HemaSphere* are international research and clinical leaders, representing a diverse range of areas of hematologic expertise.

### Open Access Licensing and Copyright

*HemaSphere* is an Open Access journal: authors of accepted articles pay an article publication charge, and their articles are published under a Creative Commons license.

Certain funders mandate a particular type of CC license is used. All other authors can choose between the CC BY-NC and CC BY-NC-ND Creative Commons Licenses.

For more information about the licenses offered by this journal, please visit Wiley Author Services.

### Disclaimer

The Publisher, European Hematology Association, and Editors cannot be held responsible for any errors in or any consequences arising from the use of information contained in this journal. The views and opinions expressed do not necessarily reflect those of the Publisher, European Hematology Association, or Editors, neither does the publication of advertisements constitute any endorsement by the Publisher, European Hematology Association, Editors, or Authors of the products advertised. Wiley Open Access articles posted to repositories or websites are without warranty from Wiley of any kind, either express or implied, including, but not limited to, warranties of merchantability, fitness for a particular purpose, or non-infringement. To the fullest extent permitted by law Wiley disclaims all liability for any loss or damage arising out of, or in connection with, the use of or inability to use the content.

HemaSphere eha

### Editorial Board

### Editor in Chief
**Professor Jan Cools**
*VIB-KU Leuven Center for Cancer Biology, Leuven, Belgium*

### Deputy Editor in Chief
**Professor Claire Harrison**
*Guy's and St Thomas' NHS Foundation Trust, London, UK*

### Associate Editors
Jeroen Eikenboom, *Leiden University Medical Centre, Leiden, The Netherlands*
Simone Ferrero, *University of Torino, Italy*
Adele Fielding, *University of York, Hull York Medical School, York, UK*
Simon Hallam, *St. Bartholomew's Hospital, Barts Health NHS Trust, London, UK*
Robert Hills, *University of Oxford, UK*
Steffen Koschmieder, *University Medical Center RWTH, Aachen, Germany*
Marek Mraz, *CEITEC Masaryk University and University Hospital Brno, Brno, Czech Republic*
Paula Rodríguez Otero, *Clínica Universidad de Navarra, Spain*
Juerg Schwaller, *University Hospital Basel, Switzerland*
Francesca Vinchi, *Iron Research Laboratory - New York Blood Center, & Weill Cornell Medicine Department of Pathology and Laboratory Medicine, New York, USA*

### Scientific Editors
Charles de Bock, *Children's Cancer Institute, Sydney, Australia*
Stephen Hibbs, *Queen Mary University of London, UK*
Edeghonghon Olayemi, *University of Ghana Medical School, Accra, Ghana*

### Editorial Manager, European Hematology Association
Jessica Mastrodomenico

### Editorial Board
Jeremy Abramson, *Massachusetts General Hospital Cancer Center, Boston, Massachusetts, USA*
Rafael Bejar, *University of San Diego, California, USA*
Marie Bene, *Centre Hospitalier Universitaire de Nantes, France*
Andrea Biondi, *University of Milano-Bicocca, Italy*
Jonathan Bond, *University College Dublin, Ireland*
Dominique Bonnet, *The Francis Crick Institute, London, UK*
Arndt Borkhardt, *University of Düsseldorf, Germany*
Dominique Bron, *Free University of Brussels, Belgium*
Christian Buske, *University of Ulm, Germany*
Suzanne Cannegieter, *Leiden University Medical Centre, The Netherlands*
Maria Domenica Capellini, *University of Milan, Italy*
Oliver Cornely, *University of Koeln, Germany*
Kim De Keersmaecker, *University of Leuven, Belgium*
Marianne De Montalembert, *Hôpital Universitaire Necker-Enfants Malades, Paris, France*
Michel Delforge, *Leuven Cancer Institute, Belgium*
Catherine Diefenbach*, Perlmutter Cancer Center, New York, USA*
Carlo Dufour, *IRCCS Istituto G. Gaslini, Genova, Italy*
Barbara Eichhorst, *University of Cologne, Germany*
Hermann Einsele, *University of Wurzbug, Germany*

Anna Falanga, *University of Milan Bicocca and Hospital Papa Giovanni XXIII, Bergamo, Italy*
Francesca Gay, *University of Turin, Italy*
Domenico Girelli, *Universita degli Studi di Verona, Italy*
Nicola Gökbuget, *University Hospital Frankfurt, Germany*
Kirsten Grønbæk, *Rigshospitalet, Copenhagen, Denmark*
Thomas Habermann, *Mayo Clinic, Rochester, Minnesota, USA*
Florian Heidel, *University Medical Center Greifswald, Germany*
Mark Hertzberg, *University of Sydney, Australia*
Brian Huntly, *University of Cambridge, UK*
Achille Iolascon, *University of Naples Federico II, Italy*
Shai Izraeli, *Schneider Children's Medical Center of Israel, Jerusalem, Israel*
Elaine Jaffe, *National Cancer Institute, Bethesda, Maryland, USA*
Efstathios Kastritis, *National and Kapodistrian University of Athens, Greece*
Marie Jose Kersten, *Academic Medical Center, Amsterdam, The Netherlands*
Jean-Jacques Kiladjian, *Hôpital Saint-Louis & Université Paris Diderot, France*
Daniela Krause, *University of Frankfurt, Germany*
Nicolaus Kröger, *University Hospital Hamburg-Eppendorf, Germany*
Ola Landgren, *Sylvester Comprehensive Cancer Center, University of Miami, Florida, USA*
Steven Lane, *QIMR Berghofer Medical Research Institute, Australia*
Maria-Victoria Mateos, *University of Salamanca, Spain*
Simon Mendez-Ferrer, *University of Cambridge, UK*
Thomas Mercher, *Gustave Roussy Institute, Villejuif, France*
Ann Mullally, *Harvard Medical School, Boston, Massachusetts, USA*
Stephen Mulligan, *Karolinska Institutet, Stockholm, Sweden*
Charlotte Niemeyer, *Albert-Ludwigs-Universität Freiburg, Germany*
Greg Nowakowski, *Mayo Clinic, Rochester, Minnesota, USA*
Esther Oliva, *Grande Ospedale Metropolitano Bianchi Melacrino Morelli, Calabria, Italy*
Jakob Passweg, *University of Basel, Switzerland*
Uwe Platzbecker, *University of Leipzig, Germany*
Marc Raaijmakers, *Erasmus Medical Center Cancer Institute, Rotterdam, The Netherlands*
David Rees, *University of London, UK*
Richard Rosenquist, *Karolinska Institutet, Stockholm, Sweden*
Davide Rossi, *Institute of Oncology Research, Bellinzona, Switzerland*
Simon Rule, *University Hospitals Plymouth NHS Trust, UK*
Rebekka Schneider, *Erasmus MC, Rotterdam, The Netherlands*
Radek Skoda, *University of Basel, Switzerland*
Kostas Stamatopoulos, *CERTH, Centre for Research and Technology Hellas, Thessaloniki, Greece*
Ulrich Steidl, *Albert Einstein College of Medicine/Montefiore Medical Center, New York, USA*
Anna Sureda, *Institut Català d'Oncologia, Barcelona, Spain*
Jan Trka, *University of Prague, Czech Republic*

### Organization

The 18th Annual Scientific Conference hosted by the Academy for Sickle Cell and Thalassaemia (ASCAT), in collaboration with the British Society of Haematology (BSH) and the European Hematology Association (EHA).

### Word of Welcome

The theme for our meeting is ‘**Therapeutic Strategies for Sickle Cell and Thalassaemia: Time to Consider A Multi-Modal & Personalised Approach**’ in recognition of the changing landscape for the management of Haemoglobinopathies. We are fully aware of the fact these new developments bring with them complex issues in decision making for patients and their families and the challenge of access to therapies It is therefore necessary to provide the platform for service providers, service users and policy-makers to engage in honest discussion to develop strategies for efficient and effective interventions across the world. Our constituency is every patient everywhere and, in all circumstances, to improve outcomes and increase access.

**The organizers would like to thank the following experts for their time and efforts reviewing abstracts for this meeting:**

Scientific Programme Committee

Abboud M, Lebanon
Andemariam B, United States
Campbell A, United States
Cappellini MD, Italy
Chakravorty S, United Kingdom
Colombatti R, Italy
de Montalembert M, France
Inusa B, United Kingdom (Chair)
Kesse-Adu R, United Kingdom
Miller R, United Kingdom
Oteng-Ntim, E, United Kingdom
Strunk, C, United States
Taher A, Lebanon
Treadwell M, United States

### CONTENTS

Oral Abstract Presentations ____ 1
Poster Abstract Presentations ____ 67
E-Poster Abstract Presentations ____ 133
